# Supplementary material for: In Vitro Influenza A Virus-Inactivating Activity of HIDROX®, Hydroxytyrosol-Rich Aqueous Olive Pulp Extract
Source: Pathogens. 2025 May 25;14(6):529. doi: 10.3390/pathogens14060529 (PMC12195756; doi:10.3390/pathogens14060529)
Supplement: Supplementary file 1 [file pathogens-14-00529-s001.zip › pathogens-3418497-supplementary.pdf]

In Vitro Influenza A Virus-Inactivating Activity of HIDROX®, Hydroxytyrosol-Rich Aqueous Olive Pulp Extract

Mayar Yasser Zeinelabideen Mohamed [1], Dulamjav Jamsransuren [2], Sachiko Matsuda [2], Koichi Narita [3], Toshihiro Murata [3], Haruko Ogawa [1] and Yohei Takeda [1,2,*]

[1] Department of Veterinary Medicine, Obihiro University of Agriculture and Veterinary Medicine, 2-11 Inada, Obihiro 080-8555, Japan; mayaryasser0@gmail.com (M.Y.Z.M.); hogawa@obihiro.ac.jp (H.O.)
[2] Research Center for Global Agromedicine, Obihiro University of Agriculture and Veterinary Medicine, 2-11 Inada, Obihiro 080-8555, Japan; jduuya@obihiro.ac.jp (D.J.); chakachaka0810@gmail.com (S.M.)
[3] Faculty of Pharmaceutical Sciences, Tohoku Medical and Pharmaceutical University, 4-4-1 Komatsushima, Aoba-ku, Sendai 981-8558, Japan; k-narita@tohoku-mpu.ac.jp (K.N.); murata-t@tohoku-mpu.ac.jp (T.M.)
* Correspondence: ytakeda@obihiro.ac.jp; Tel.: +81-155-49-5896

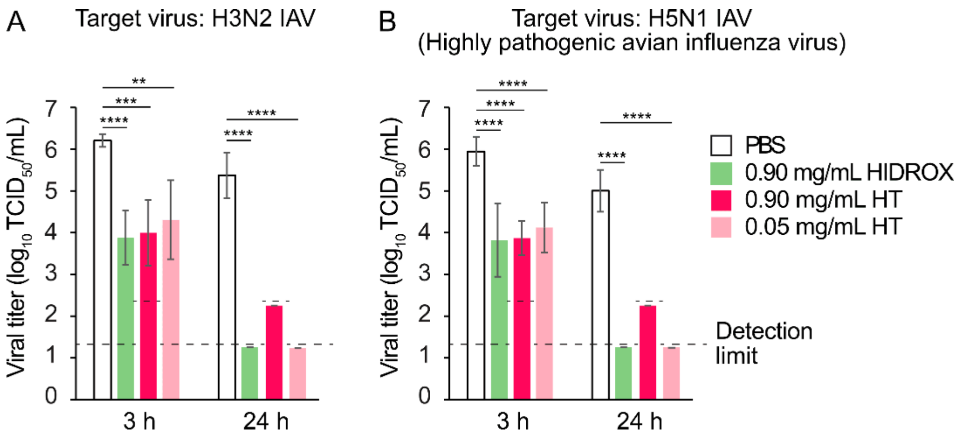

Supplementary Figure S1. Virucidal activity of HIDROX and HT against multiple subtype IAVs. Unpurified H3 (A) or H5 (B) IAV solution was mixed with PBS, HIDROX, or HT solution. Mixtures were incubated at 25 °C for 3 and 24 h. The titer in each group is shown. The detection limits (dashed line) of the viral titer were $10^{1.25}$ $TCID_{50}$/mL in the PBS, 0.90 mg/mL HIDROX, and 0.05 mg/mL HT groups, and $10^{2.25}$ $TCID_{50}$/mL in the 0.90 mg/mL HT group. Results are indicated as mean ± SD ($n = 8$ per group). (A) Kruskal–Wallis test followed by Dunn’s multiple comparisons test was performed. (B) One-way ANOVA followed by Tukey´s multiple comparisons test (for 3 h), and Kruskal–Wallis test followed by Dunn’s multiple comparisons test (for 24 h), were performed; $^{**}p < 0.01$; $^{***}p < 0.001$; $^{****}p < 0.0001$.

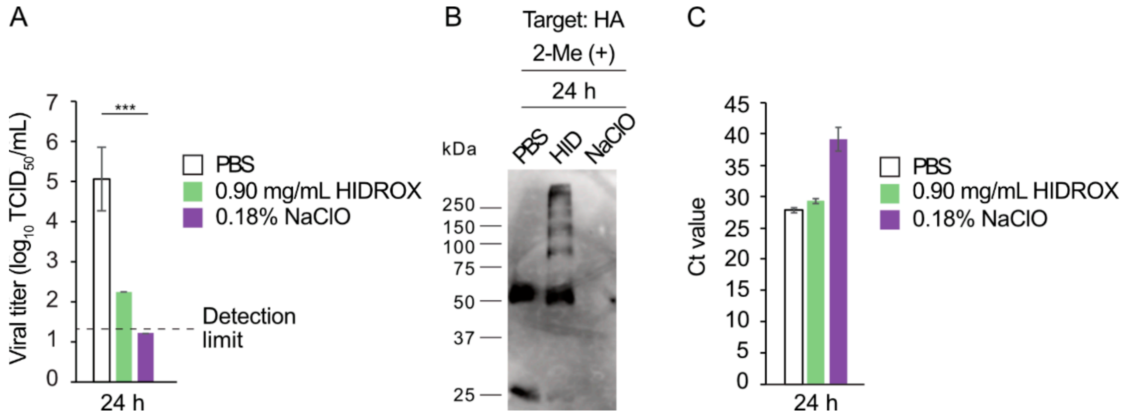

Supplementary Figure S2. Impact of HIDROX and NaClO solution treatments on IAV. (A) Purified H1 IAV solution was mixed with PBS, HIDROX, or NaClO solution, and the mixtures were incubated at 25 °C for 24 h. The viral titer in each group is shown. The detection limits (dashed line) of the viral titer were $10^{1.25}$ $TCID_{50}$/mL. Results are indicated as mean ± SD (n = 6 per group). Kruskal–Wallis test followed by Dunn's multiple comparisons test was performed; ***$p < 0.001$. (B) Purified H1 IAV solution was mixed with PBS, HIDROX, or NaClO solution. Mixtures were incubated at 25 °C for 24 h. The image is the result of WB for HA1 and HA2. HID: HIDROX. (C) Purified H1 IAV solution was mixed with PBS, HIDROX, or NaClO solution. Mixtures were incubated at 25 °C for 24 h. Real-time RT-PCR targeting the IAV M gene was performed, and the Ct value was evaluated. Results are indicated as mean ± SD (n = 2 per group).

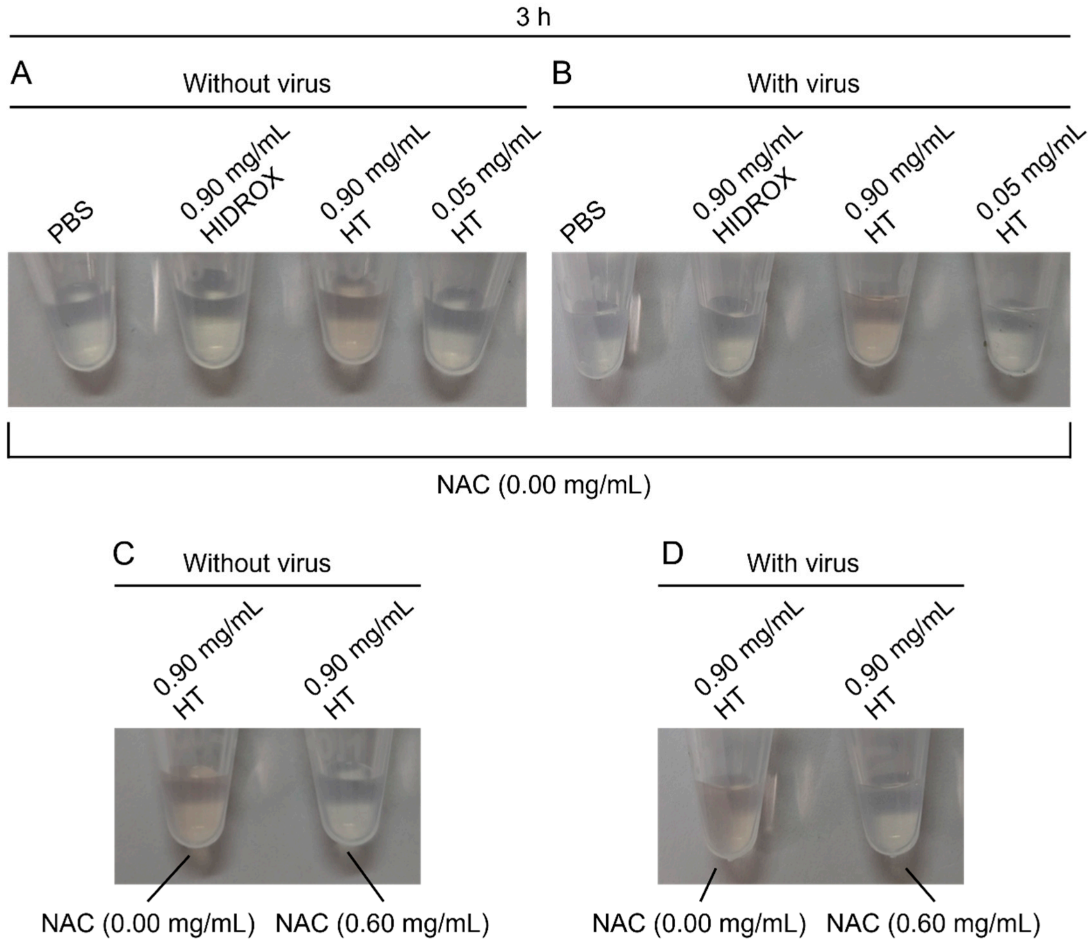

Supplementary Figure S3. Photographs showing the color of various solutions containing HIDROX and HT following incubation for 3 h at 25 °C. (A) PBS, HIDROX/PBS, and HT/PBS solutions (without the virus) in the absence of NAC. (B) Inactivated/dialyzed H1 IAV solutions mixed with PBS, HIDROX, and HT solutions in the absence of NAC. (C) HT/PBS in the absence and presence of NAC. (D) Inactivated/dialyzed H1 IAV solutions mixed with HT in the absence and presence of NAC.
